# Supplementary material for: Adverse caregiving in infancy blunts neural processing of the mother
Source: Nat Commun. 2020 Feb 28;11:1119. doi: 10.1038/s41467-020-14801-3 (PMC7048726; doi:10.1038/s41467-020-14801-3)
Supplement: Supplementary file 2 — Reporting Summary [file 41467_2020_14801_MOESM2_ESM.pdf]

nature research

Corresponding author(s): Maya Opendak

Last updated by author(s): Sep 10, 2019

Reporting Summary

Nature Research wishes to improve the reproducibility of the work that we publish. This form provides structure for consistency and transparency in reporting. For further information on Nature Research policies, see Authors & Referees and the Editorial Policy Checklist .

Statistics

For all statistical analyses, confirm that the following items are present in the figure legend, table legend, main text, or Methods section.

n/a	Confirmed	
☐	☒	The exact sample size (n) for each experimental group/condition, given as a discrete number and unit of measurement
☐	☒	A statement on whether measurements were taken from distinct samples or whether the same sample was measured repeatedly
☐	☒	The statistical test(s) used AND whether they are one- or two-sided<br>*Only common tests should be described solely by name; describe more complex techniques in the Methods section.*
☐	☒	A description of all covariates tested
☐	☒	A description of any assumptions or corrections, such as tests of normality and adjustment for multiple comparisons
☐	☒	A full description of the statistical parameters including central tendency (e.g. means) or other basic estimates (e.g. regression coefficient) AND variation (e.g. standard deviation) or associated estimates of uncertainty (e.g. confidence intervals)
☐	☒	For null hypothesis testing, the test statistic (e.g. F, t, r) with confidence intervals, effect sizes, degrees of freedom and P value noted<br>*Give P values as exact values whenever suitable.*
☒	☐	For Bayesian analysis, information on the choice of priors and Markov chain Monte Carlo settings
☒	☐	For hierarchical and complex designs, identification of the appropriate level for tests and full reporting of outcomes
☒	☐	Estimates of effect sizes (e.g. Cohen's d, Pearson's r), indicating how they were calculated

Our web collection on statistics for biologists contains articles on many of the points above.

Software and code

Policy information about availability of computer code

Data collection	Data collection for LFP using Spike2 software, Behavior scored using BORIS
Data analysis	Data analysis for FP using Spike2 software, LFP circularity analysis via MATLAB

For manuscripts utilizing custom algorithms or software that are central to the research but not yet described in published literature, software must be made available to editors/reviewers. We strongly encourage code deposition in a community repository (e.g. GitHub). See the Nature Research guidelines for submitting code & software for further information.

Data

Policy information about availability of data

All manuscripts must include a data availability statement. This statement should provide the following information, where applicable:

- Accession codes, unique identifiers, or web links for publicly available datasets
- A list of figures that have associated raw data
- A description of any restrictions on data availability

The data that support the findings of this study are available from the corresponding author upon request. We have included a Source Data File for all relevant figures in the manuscript.

Field-specific reporting

Please select the one below that is the best fit for your research. If you are not sure, read the appropriate sections before making your selection.

☒ Life sciences ☐ Behavioural & social sciences ☐ Ecological, evolutionary & environmental sciences

Life sciences study design

All studies must disclose on these points even when the disclosure is negative.

Sample size	Based on previous studies with oscillations in pups, we estimated effect sizes expected to be obtained and subsequently conducted a power analysis to determine the minimum number of animals required per group to have sufficient statistical power to detect differences between our experimental groups.
Data exclusions	Animals that failed to thrive after surgical implantation were excluded from further analyses. Behavioral scoring of maternal behavior was used to validate whether or not mothers given low bedding were maltreating pups and litters were excluded on this basis.
Replication	All behavior was done with lower n as pilot data and then replicated for formal study (including those with pharmacology and mom manipulations).
Randomization	Pups from each litter were counterbalanced via random assignment into rearing groups and pharmacological CORT inhibition, such that all data collection and processing was conducted randomly. To control for litter effects, a maximum of 1 male and 1 female were used from each litter per experimental condition.
Blinding	All Strange Situation Procedure neurobehavioral data (LFP and behavior) was analyzed blind to pup rearing/drug conditions. All LFP data within the nest was analyzed by experimenters blind to the rearing condition.

Reporting for specific materials, systems and methods

We require information from authors about some types of materials, experimental systems and methods used in many studies. Here, indicate whether each material, system or method listed is relevant to your study. If you are not sure if a list item applies to your research, read the appropriate section before selecting a response.

Materials & experimental systems

n/a	Involved in the study	
☒	☐	Antibodies
☒	☐	Eukaryotic cell lines
☒	☐	Palaeontology
☐	☒	Animals and other organisms
☐	☒	Human research participants
☒	☐	Clinical data

Methods

n/a	Involved in the study	
☒	☐	ChIP-seq
☒	☐	Flow cytometry
☒	☐	MRI-based neuroimaging

Animals and other organisms

Policy information about studies involving animals; ARRIVE guidelines recommended for reporting animal research

Laboratory animals	Rattus norvegicus, Long Evans strain, males and females aged 8- 14 days old.
Wild animals	No wild animals were used in this study
Field-collected samples	No field collected animals were used in this study
Ethics oversight	Nathan S. Kline Institute Institutional Animal Care and Use Committee

Note that full information on the approval of the study protocol must also be provided in the manuscript.

Human research participants

Policy information about studies involving human research participants

Population characteristics	Risk factors in children.<br>Cumulative risk indices. Cumulative risk indices were developed across three domains: child, parent, instability, consistent with previous literature 15,16. Information for the cumulative risk indices were obtained from demographic questionnaires completed by parents, as well as questionnaires regarding parental mental health and child temperament. In addition, a life events interview utilizing a calendar-based method was conducted with the parent. This interview queried the presence of a range of risk factors throughout the child's life. Information from all sources was consolidated. Each risk factor was given a score of zero (0) if absent and a score of one (1) if present, and the total number of risk factors was summed for each child. Risk factors were assessed as present or absent during the child's first two years. Information regarding specific risk factors are included below. See Table S4 for descriptive information regarding risk factors for each group. The high-risk group had a significantly higher cumulative risk total score (M = 8.7, SD = 1.7) than the low-risk group (M = 2.8, SD = 1.2; t(19) = -9.15, p

< .001).
Child cumulative risk.
Child low birth weight. Parents reported the child's birth weight on the demographic questionnaires. Children who were reported to have a birth weight greater than 2500 gm were given a score of zero (0), whereas those reported to have a birth weight less than 2500 gm were given a score of one (1). Low birth weight (LBW) is defined by the World Health Organization (WHO) as weight at birth less than 2500 gm17.
Prenatal substance or alcohol exposure. Parents reported prenatal alcohol or substance use on demographic questionnaires. In addition, parents were asked about their alcohol and substance use on the life events interview, and when they learned that they were pregnant. Children whose parents reported using no alcohol or substances during pregnancy were given a score of zero (0), whereas those whose parents reported using alcohol or substances during pregnancy were given a score of one (1).
Difficult temperament. Parents completed the Infant Behavior Questionnaire - Revised (IBQ-R18), which is designed to assess temperament in young children. Two scales were used to assess children's temperament: the Distress to Limitations scale, which measures the child's reactions to limitations such as delays in feeding and being placed in a confining position such as a car seat, and the Soothability scale (reversed scored), which assesses the child's reduction of fussing, crying, or distress when the parent uses soothing techniques. Children whose parents scored them less than one standard deviation above the average were given a score of zero (0), whereas children whose parents scored them more than one standard deviation above the average were given a score of one (1).
Parent cumulative risk.
Income. On the demographic questionnaires, the parent reported yearly family income from all sources (e.g., employment, child support, Temporary Assistance for Needy Families [TANF], etc.). The number of family members living in the residence was also reported on the demographic questionnaires and through the life events interview. An income-to-needs ratio was calculated using this information based on the poverty guidelines which updated periodically in the Federal Register by the U.S. Department of Health and Human Services under the authority of 42 U.S.C. 9902. Parents who reported a ratio greater than 1.0 (above the poverty line) were given a score of zero (0), while those who reported a ratio lower than 1.0 (below the poverty line), were given a score of one (1).
Parent mental health. Parent mental health was measured using the Psychiatric Diagnostic Screening Questionnaire (PDSQ), a 125 item self-report checklist designed to assess the presence of psychopathological symptoms 19. Parents who reported symptoms that exceeded the "clinical cutoff" score for depression, anxiety, or posttraumatic stress disorder on the PDSQ or who reported significant mental health concerns on a life events interview were given a score of one (1). Those who did not report significant mental health concerns on the PDSQ or interview were given a score of zero (0).
Parent education. Parents completed demographic information forms listing their education level. Parents who completed high school or beyond (or obtained a GED) were given a score of zero (0). Those who did not complete high school were given a score of one (1).
Parent employment. Demographic forms were completed by parents and the life events interview assessed periods of employment. Parents who were employed during the relevant time period were given a score of zero (0), whereas those who were not employed were given a score of one (1).
Parent criminal justice involvement. The life events interview and the demographic questionnaires inquired about parental involvement with the criminal justice system. Parents with no criminal justice system involvement during the relevant time period were given a score of zero (0), whereas those with criminal justice system involved during the time period were given a score of one (1).
Age first became parent. Demographic questionnaires completed by parents at the first time point inquired about the parent's date of birth, as well as the dates of birth of all of their children. Based on this information, the age at which parent first gave birth was calculated. Parents who were 18 years or older when they first became a parent were given a score of zero (0), whereas those who were 17 years old or younger when they first became a parent were given a score of one (1).
Single parent. Information regarding the parent's marital and relationship status was collected through demographic questionnaires, and interview questions regarding the presence of a partner during the time period. Parents who reported having a partner for 50% or more of the relevant time period were given a score of zero (0), whereas those who reported not having a partner for more than 50% of the time period were given a score of one (1).
Parent substance abuse. The demographic questionnaire given at the first time period assessed for substance abuse, and parents were also asked about a history of substance abuse during the life events interview. Parents who reported no substance abuse during the relevant time period were given a score of zero (0), whereas those who reported substance abuse during the time period were given a score of one (1).
Instability cumulative risk.
Residential moves. During the life events interview the parent described each residence since the child was born and when residential moves occurred. If no residential moves occurred during the relevant time period, the parent was given a score of zero (0). Parents who reported residential moves occurring during the relevant time period (when the child was 0 to 2 years old) were given a score of one (1).
Changes in romantic partners. Parents reviewed their romantic relationships during the life events interview, including periods when relationships began or ended. If the parent reported no changes in romantic relationships during the relevant time period, the parent was given a score of zero (0). Parents who reported the beginning or end of a romantic relationship during the relevant time period were given a score of one (1).
Homelessness. Information regarding homelessness was gathered from the initial referral information, as well as through the interview utilizing a calendar method. Parents who reported no homelessness during the relevant time period were given a score of zero (0). Those who reported being homeless at any time during the relevant time period were given a score of one (1).
Significant separations between child and parent. Parents described any significant separations (for one month or more) between child and parent in the life events interview. Parents who reported no significant separations during the relevant time period were given a score of zero (0), whereas those that reported a significant separation during the time period were given a score of one (1).
Removal of other children by CPS. Parents reported on the removal of other children by CPS on the first demographic form, and also in the life events interview. Parents who reported no children removed by CPS were given a score of zero (0), whereas parents who reported other children removed by CPS were given a score of one (1).

Recruitment

Parents and their children were referred to the study because of risks for maltreatment, such as unstable living conditions, having mental health or substance abuse problems, or evidence of prior maltreatment. From this larger sample, two smaller samples were selected: a high-risk group that had at least 6 total risk factors and a control group that had no more than 5 or fewer total risk factors. Please see Tables S3 and S4 for additional information regarding human participants.
